# Assessment of canine *BEST1* variations identifies new mutations and establishes an independent bestrophinopathy model (*cmr3*)

**Published:** 2010-12-16

**Authors:** Barbara Zangerl, Kaisa Wickström, Julianna Slavik, Sarah J. Lindauer, Saija Ahonen, Claude Schelling, Hannes Lohi, Karina E. Guziewicz, Gustavo D. Aguirre

**Affiliations:** 1 Section of Ophthalmology, School of Veterinary Medicine, University of Pennsylvania, Philadelphia, PA; 2 Veterinary Clinic, Akuutti, Finland; 3 Department of Veterinary Biosciences, Department of Medical Genetics, Program in Molecular Medicine, University of Helsinki and Folkhälsan Research Institute, Helsinki, Finland; 4 Department of Animal Sciences, Swiss Federal Institute of Technology Zurich and Vetsuisse Faculty Zurich, University of Zurich, Zurich, Switzerland

## Abstract

**Purpose:**

Mutations in bestrophin 1 (*BEST1*) are associated with a group of retinal disorders known as bestrophinopathies in man and canine multifocal retinopathies (*cmr*) in the dog. To date, the dog is the only large animal model suitable for the complex characterization and in-depth studies of Best-related disorders. In the first report of *cmr*, the disease was described in a group of mastiff-related breeds (*cmr1*) and the Coton de Tulear (*cmr2*). Additional breeds, e.g., the Lapponian herder (LH) and others, subsequently were recognized with similar phenotypes, but linked loci are unknown. Analysis of the *BEST1* gene aimed to identify mutations in these additional populations and extend our understanding of genotype–phenotype associations.

**Methods:**

Animals were subjected to routine eye exams, phenotypically characterized, and samples were collected for molecular studies. Known *BEST1* mutations were assessed, and the canine *BEST1* coding exons were amplified and sequenced in selected individuals that exhibited a *cmr* compatible phenotype but that did not carry known mutations. Resulting sequence changes were genotyped in several different breeds and evaluated in the context of the phenotype.

**Results:**

Seven novel coding variants were identified in exon 10 of c*BEST1*. Two linked mutations were associated with *cmr* exclusive to the LH breed (*cmr3*). Two individuals of Jämthund and Norfolk terrier breeds were heterozygous for two conservative changes, but these were unlikely to have disease-causing potential. Another three substitutions were found in the Bernese mountain dog that were predicted to have a deleterious effect on protein function. Previously reported mutations were excluded from segregation in these populations, but *cmr1* was confirmed in another mastiff-related breed, the Italian cane corso.

**Conclusions:**

A third independent canine model for human bestrophinopathies has been established in the LH breed. While exhibiting a phenotype comparable to *cmr1* and *cmr2*, the novel *cmr3* mutation is predicted to be based on a distinctly different molecular mechanism. So far *cmr2* and *cmr3* are exclusive to a single dog breed each. In contrast, *cmr1* is found in multiple related breeds. Additional sequence alterations identified in exon 10 of c*BEST1* in other breeds exhibit potential disease-causing features. The inherent genetic and phenotypic variation observed with retinal disorders in canines is complicated further by *cmr3* being one of four distinct genetic retinal traits found to segregate in LH. Thus, a combination of phenotypic, molecular, and population analysis is required to establish a strong phenotype–genotype association. These results indicate that *cmr* has a larger impact on the general dog population than was initially suspected. The complexity of these models further confirms the similarity to human bestrophinopathies. Moreover, analyses of multiple canine models will provide additional insight into the molecular basis underlying diseases caused by mutations in *BEST1*.

## Introduction

A major challenge in the description, interpretation, and therapy for genetically caused diseases is identifying unique phenotypic disease characteristics and discerning their underlying genotype. On one hand, genetic heterogeneity can result in the same phenotype in a single population, which is caused by mutations in one (allelic heterogeneity) or more (nonallelic heterogeneity) loci. On the other hand, individual loci or genes can be associated with several distinctly different disorders, commonly known as a pleiotropic effect. Both phenomena are common to blinding disorders. For example, a single retinal phenotype, such as retinitis pigmentosa, is linked to several genes and loci (>40 loci, RetNet). Conversely, alterations in frequently mutated genes in humans, e.g., retinal-specific ATP-binding cassette transporter (*ABCA4*) or bestrophin 1 (*BEST1*), result in pleiotropic effects and are associated with large numbers of overlapping phenotypes [1-4]. Diagnoses are further complicated as, in many cases, the disease-causing mutation and modifying alleles are not yet known. Mutations in the *BEST1* (also known as *VMD2*) gene cause several human retinal disorders grouped as bestrophinopathies [5-7]. Despite differences in the clinical appearance and mode of inheritance between individual patients, these disorders predominantly affect the macula and fovea area, with varying involvement of the peripheral retina having been reported [8-10].

Autosomal dominant Best vitelliform macular dystrophy (BVMD), the most prominent bestrophinopathy phenotype, typically presents in childhood with the appearance of a single, yolk-like lesion of the macula. However, variation occurs in the disease manifestation, which can extend to multifocal vitelliform, atrophic lesions, or chorioretinal scars. Additionally, the age of onset and progression within individual pedigrees is not consistent. As a consequence, visual impairment in patients ranges from minor dropout of central vision to complete central blindness following atrophy of the retinal pigment epithelium and photoreceptor degeneration [10-12]. Overlapping clinical presentation between BVMD and other bestrophinopathies [4], caused by well over 100 different mutations in *BEST1*, demonstrates the complexity of genotype–phenotype associations. These intricacies hamper development of allele- and phenotype-independent therapy options. The lack of a clear prognosis and of treatment options adds to the emotional strain for children and young adults with early onset bestrophinopathies.

The development of large animal models complements ongoing research in rodents, the latter of which do not necessarily recapitulate the complexity of the human phenotype [13]. The particular population structure of the dog is a direct result of separating isolated breeds for many generations and proves advantageous for tracking phenotypic and disease traits [14,15]. Identification of mutations causing canine blinding disorders has not only improved breeding strategies but has also significantly advanced the understanding of disease mechanisms and development of therapies [16]. To this end, several experimental studies in dogs have formed the basis for human clinical trials, and many more are expected to follow suit [17-19]. As the molecular bases of inherited retinal disorders are being discovered, it appears that simple mutation–breed associations are not necessarily the rule. While these do occur [20,21], one cannot predict the number of breeds associated with either allelic [22] or nonallelic [23,24] forms of disease.

Two mutations in canine *BEST1* (*cBEST1*) have previously been described in different dog breeds and established the canine bestrophinopathy models [24]. Both the C73T premature stop mutation (R25X) in Great Pyrenees and mastiff-related breeds, and the G482A missense mutation (G161D) in the Coton de Tulear present with a characteristic clinical manifestation termed canine multifocal retinopathy (*cmr*). The autosomal recessive, post-developmental disease phenotype results in early onset of retinal elevations, with areas containing subretinal pink-tan fluid that develop into focal to multifocal outer retinal atrophy. Similar lesions have been recognized in other canine populations, but thus far the responsible genetic defects are unknown. One of these breeds, the Lapponian herder (LH), native to Finland, is known to also be affected by progressive rod–cone degeneration (*prcd*) [22,25], the most common retinal degeneration in dogs distributed in >20 distinct breeds. However, after eliminating *prcd*-affected animals, phenotypic variation in LH with retinal abnormalities remained, with pathologies including lesions mimicking *cmr*, multifocal retinal dysplasia (MRD), post-inflammatory changes, and generalized progressive retinal atrophy (PRA). Based on the onset of the multifocal retinopathy at approximately 1 year of age, and similarities in the disease manifestation to *cmr*, we hypothesized that at least a subset of the spectrum of retinal diseases in LH could be explained by mutations in *cBEST1*.

To examine the association of known and new *cBEST1* mutations in LH and other breeds, a total of 614 dogs from 38 different breeds were assessed for *cmr1* and *cmr2* mutations (Table 1). The presence of the *cmr1* mutation was confirmed in additional mastiff-related dog populations, including the Italian cane corso (also known as Italian mastiff), but was not present in a large LH pedigree or isolated cases of other breeds. Notably, seven novel coding changes in *cBEST1* exon 10 were recognized in those samples. Two of these were associated with one (*cmr3*) of four different retinal diseases in the LH. The presented variation among and within individual *cmr* genotypes appears comparable to human BVMD [5]. Thus, the different molecular consequences and independent breed backgrounds of the canine bestrophinopathy models will provide a solid basis for investigating genotype–phenotype correlations with regard to *BEST1* mutations and, most importantly, present insights for potential therapeutic intervention.

**Table 1:** Dog breeds screened for *cBEST1*.

**Evaluated**	**Breed**	**#**	**mutation/disease**
*cBEST1* coding region	Karelian bear dog	2	
	Kuvasz	1	
	Lapponian herder	5	P463fs
			G489V
	Swedish vallhund	2	
*cBEST1* exon 10	Affenpinscher	1	
	Akita	1	
	Basenji	4	
	Belgian shepherd dog	1	
	Bernese mountain dog	135	K438R
			W440C
			V473G
	Border collie	1	
	Cavalier King Charles Spaniel	1	
	Great dane	1	
	Havanese	1	
	Ibizan hound	1	
	Jämthund	1	T505S
	Karelian bear dog	4	
	Miniature spitz	2	
	Lancashire heeler	1	
	Löwchen	1	
	Miniature schnauzer	2	
	Norfolk terrier	1	W440L
	Norrbottenspetz	1	
	Parson russel terrier	5	
	Rottweiler	1	
	Russian hound	1	
	Tibetan spaniel	2	
*cmr* mutations	Affenpinscher	1	
	Akita	1	
	Argentinean mastiff	19	
	Basenji	4	
	Belgian shepherd dog	1	
	Bernese mountain dog	392	
	Border collie	1	
	Cavalier King Charles Spaniel	1	
	Dogue de Bordeaux	30	*cmr1*
	English mastiff	2	*cmr1*
	Finnish lapphund	1	
	German shorthaired pointer	4	
	Great dane	1	
	Havanese	1	
	Ibizan hound	1	
	Irish setter	5	
	Italian cane corso	44	*cmr1*
	Jämthund	1	
	Karelian bear dog	6	
	Miniature spitz	2	
	Kuvasz	1	
	Lancashire heeler	1	
	Lapponian herder	54	*cmr3*
	Löwchen	1	
	Miniature schnauzer	2	
	Neapolitan mastiff	2	
	Newfoundland	3	
	Norfolk terrier	1	
	Norrbottenspetz	1	
	Norwegian elkhound	2	
	Parson russel terrier	5	
	Pyrenean mastiff	7	
	Rottweiler	1	
	Russian hound	1	
	Samoyed	4	
	Spanish mastiff	6	
	Swedish vallhund	2	
	Tibetan spaniel	2	

A total of 179 individuals from 25 different breeds diagnosed with retinopathy, retinal degenerations, or unspecified retinal changes were sequenced for either the complete *cBEST1* coding region or exon 10 only. In addition to two mutations (P463FS, G489V) identified in *cmr3* affected Lapponian herder, three novel polymorphisms (Table 2) and five heterozygote sequence variations were observed. Subsequently, all 614 dogs from 38 breeds enrolled in the study were evaluated for *cmr1*, *cmr2*, and *cmr3* mutations.

## Methods

### Sample collection

Blood in anticoagulant or buccal swabs were obtained from privately owned dogs in accordance with standard clinical veterinary care and forwarded to research laboratories as part of ongoing research at the University of Pennsylvania, the University of Helsinki and Folkhälsan Research Center, or the University of Zurich. DNA was extracted using the DNeasy Blood & Tissue Kit (Qiagen Inc., Valencia, CA) following the manufacturer’s protocol and stored at −80 °C until further use. After exclusion of other known retinal disorders in the respective breeds (*prcd* [22]; X-linked PRA [23]; Collie Eye Anomaly [26]), 614 samples from 38 individual breeds were enrolled in the current study as normal controls and dogs with retinal disease of unknown cause (Table 1).

### Phenotype evaluation

Except for most Bernese mountain dogs (BMD) and mastiffs, all dogs received routine ophthalmic examination following the guidelines of the Canine Eye Registration Foundation, American College of Veterinary Ophthalmologists, and the European College of Veterinary Ophthalmology hereditary eye disease scheme. All LH in the study were phenotypically evaluated by one of the co-authors (KW); this represented a total of 70 animals based on data covering more than 10 years. However, DNA was available from only 54 of these dogs. Dogs included in the genetic study were examined between 1 and 2 years of age in most cases; follow-up exams were scheduled every 1–2 years depending on findings. Although *cmr* lesions develop before 2 years of age, only dogs with no changes after 3 years of age were considered nonaffected. These stringent criteria identified a total of eight *cmr*-affected LH and 46 that were either normal or had other fundus changes.

### *BEST1* sequence comparison

All coding exons and flanking splice junctions of c*BEST1* (exons 2 through 11) were amplified individually from dogs with clinical disease similar to *cmr* and three obligate carrier LH dogs (Table 1), using gene-specific primers and conditions previously published [24]. Amplification products were visualized on 1% agarose gels and purified using the QIAquick Gel Extraction Kit (Qiagen Inc., Valencia, CA). Sequences were obtained by direct sequencing of the direct and indirect strand (ABI 3730 sequencer; Applied Biosystems, Foster City, CA) at the DNA Sequencing Facility of the University of Pennsylvania and evaluated with the Sequencher 4.2.2 software package (Gene Codes Corporation, Ann Arbor, MI).

Additional breeds harboring molecularly undefined retinal changes were selected based on a potentially shared ancestral or geographic origin with LH (Table 1). From these, c*BEST1* Exon 10 was amplified under the same conditions; forward primer 5′- AAG GAG GGA AAA GAT AGG GT −3′, reverse primer 5′- AGG TGG AAG GAG GGT AGA AT −3′), and sequenced in both directions (forward primer 5′- CTC ACC CAG GTG TGT GTT TG −3′, reverse primer 5′- TCA AGT CCT GCT TTG GTC CT −3′). All obtained sequences were aligned against the canine genome sequence draft [27] and *cBEST1* reference sequence (NM_001097545). Observed sequence alterations were analyzed for their potential to affect protein function using the Sorting Intolerant From Tolerant (SIFT) algorithm [28,29].

### Mutation screening and testing

#### Allele-specific PCR amplification

Confirmed *cmr* mutations were tested in all enrolled dogs (Table 1). Tests for *cmr1* (C73T/R25X) and *cmr2* (G482A/G161D) mutations followed published protocols [24]; for the *cmr3* sequence variations, tests were established for amplification of the wild type (WT) or mutation (LH)-specific allele. Two allele-specific primers for C1388del (WT: 5′-AGG CTA CCA CAG TGC CCC A-3′, LH: 5′-CAG GCT ACC ACA GTG CCC A-3′) were amplified against an anchor primer (5′-CTC ACC CAG GTG TGT GTT TG-3′), using 1.5 mM MgCl_2_, 0.4 µM of each primer, 0.2 µM of each dNTP, and 0.875 U Taq polymerase (Invitrogen, Carlsbad, CA) on 50 ng of genomic DNA in 35 cycles at 94 °C for 45 s, 69 °C for 30 s, and 72 °C for 30 s, after an initial denaturation at 94 °C for 1 min and followed by a final extension at 72 °C for 10 min. The 420-bp PCR product was separated on 6% polyacrylamide gels and scored based on the presence or absence of DNA product. The G1466T test used the same anchor primer combined with primers specific to the respective alleles (WT: 5′-CCT ACG CAG AGT CTC AGG G-3′, LH: 5′-CCT ACG CAG AGT CTC AGT G-3′). Amplification and genotyping of the 342-bp PCR product was obtained and evaluated under the same conditions as *cmr3*, substituting the annealing temperature for 68 °C (WT) and 67 °C (LH), respectively.

#### Restriction enzyme digest

Coding changes identified in the BMD were screened in all individuals of the breed by either direct sequencing, as described above (*BEST1* sequence comparison), or restriction enzyme tests. Thus, c*BEST1* exon 10 was amplified and subsequently digested at 37 °C overnight with StuI, BstNI, and BbsI (New England Biolabs, Ipswich, MA) to assess the Lys438Arg, Trp440Cys, and Val473Gly substitutions, respectively. Resulting DNA patterns were evaluated on 1% agarose gels reflecting the a) Lys438Arg WT allele (A) by 304- and 521-bp bands versus the 825-bp mutant allele (G); b) Trp440Cys WT allele (G) by 36- and 185-bp bands compared to a 121-bp allele for the mutant allele (T) in addition to 38-, 54-, 84-, 186-, and 242-bp bands present with both genotypes; c) Val473Gly WT allele (T) by 100- and 426-bp bands in contrast to a 526-bp band indicating the mutant allele (G) next to 107- and 192-bp products being present with both genotypes.

#### Intron 2 and 3 polymorphism screening

Two polymorphisms in introns 2 and 3 (Table 2, #2 and #3) were assessed in LH by restriction enzyme digest. Single nucleotide polymorphism (SNP) 57,507,851 (C/T) was located within a 346-bp PCR product (primers 5′-ACT TAT GAG GCC CAG ACA AGC-3′ and 5′-TGA ATG GCT GGC TAT TTG TTC-3′) obtained from 50 ng genomic DNA with 1.5 mM MgCl_2_, 0.625 µM of each primer, 0.2 µM of each dNTP, and 0.875 U Qiagen Taq polymerase in 35 cycles at 60 °C annealing temperature. The PCR product was digested with 20 U of Eco01091 (New England Biolabs) at 37 °C for 1 h, resulting in a diagnostic product of either 213 bp (C) or 259 bp (T) in size. Similarly, SNP 57,506,423 (C/T) was included in a 561-bp PCR product (primers 5′-GTG TGC TCC CAG TGT CTA CAT C-3′ and 5′-CAC GAC CAG AGT CAC GTA GAA G-3′), using the same conditions as described above at 65 °C annealing temperature. Digestion was achieved with 20 U of AatII (New England Biolabs) at 37 °C for 2 h. The presence of 237- and 314-bp bands distinguished the common allele (C) from the minor allele (T) which had 259- and 302-bp products.

**Table 2:** Observed genomic variation in the c*BEST1* gene.

**Number**	**CFA18 bp**	**Gene position**	**Variation**	**Consequence**	**Observed in**	**Reference**
1	57507965	Exon 2	G>A	silent	Karelian beardog (KBD)	[24]
2	58507851	Intron 2	C>T	non-coding	Lapponian herder	BICF2P299043^2^
3	57506423	Intron 3	C>T	non-coding	three or more breeds	[24]
4	57506082	Exon 4	C>A	silent	three or more breeds	[24]
5	57505871	Intron 4	G>A	non-coding	Swedish vallhund (SV)	[24]
6	57505579	Intron 4	A>G	non-coding	Kuvasz	[24]
7	57505571	Intron 4	A>G	non-coding	KBD, SV	BICF2G630689281^2^
8	57505345	Intron 5	C>T	non-coding	Kuvasz	[24]
9	57505321	Intron 5	T>C	non-coding	Kuvasz	[24]
10	57505315	Intron 5	C>A	non-coding	Kuvasz	[24]
11	57504881	Intron 5	C>G	non-coding	Swedish vallhund	[24]
12	57504859	Intron 5	A>G	non-coding	KBD, SV	NCBI ss250608388
13	57504620	Intron 6	G>A	non-coding	three or more breeds	BICF2G630689283^2^
14	57504059	Intron 6	T>A	non-coding	Swedish vallhund	BICF2G630689286^2^
15	57503846	Exon 7	G>A	silent	three or more breeds	BICF2G630689287^2^
16	57503789	Intron 7	A>G	non-coding	three or more breeds	BICF2G630689288^2^
17	57502488	Intron 7	A>G	non-coding	Swedish vallhund	BICFPJ1456205^1^
18	57502387	Exon 8	T>C	silent	Karelian beardog	NCBI ss250608389
19	57502384	Exon 8	T>C	silent	KBD, SV	BICFPJ1456206^1^
20	57500034	Exon 10	A>G	Lys438Arg	Bernese mountain dog	NCBI ss250608394
21	57500028	Exon 10	G>T	Trp440Leu	Norfolk terrier	NCBI ss250608395
22	57500027	Exon 10	G>T	Trp440Cys	Bernese mountain dog	NCBI ss250608396
**23**	**57499959**	**Exon 10**	**C>del**	**Pro463fs**	**Lapponian herder**	**NCBI**ss250608397
24	57499929	Exon 10	T>G	Val473Gly	Bernese mountain dog	NCBI ss250608398
**25**	**57499881**	**Exon 10**	**G>T**	**Gly489Val**	**Lapponian herder**	**NCBI**ss250608399
26	57499834	Exon 10	A>T	Thr505Ser	Jämthund	NCBI ss250608400
27	57449754	Exon 10	C>T	silent	three or more breeds	NCBI ss250608401
28	57498425	3′UTR	A>T	non-coding	Swedish vallhund	[24]

The majority of polymorphisms identified had previously been reported. Three new polymorphisms (row #12, 18, and 27), however, were observed in addition to seven coding changes in exon 10 (row #20–26); two of these segregate with retinal disease in the Lapponian herder breed (row #23 and 25, **bold,** *cmr3*). *CFA18=*Canis familiaris* chromosome 18. ^1^Based on CanFam1;^2^Based on Can Fam2.

## Results

### Retinal phenotypes

**Figure 1:**
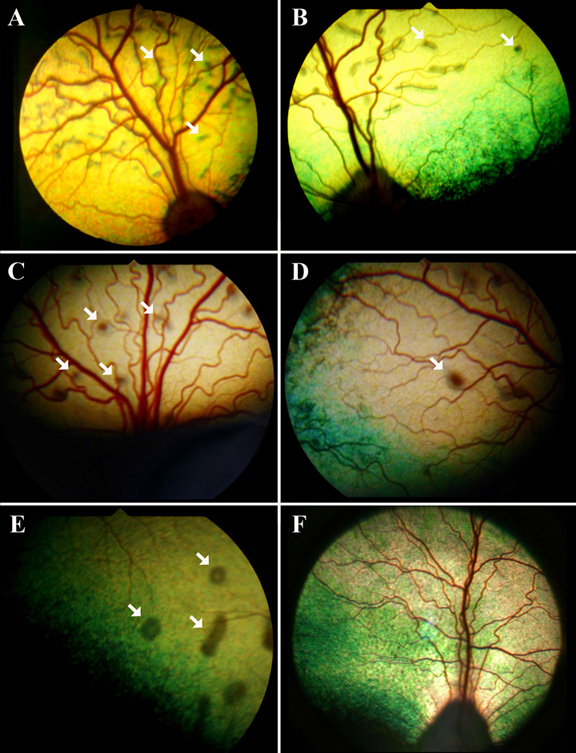
Multifocal retinal dysplasia and canine multifocal retinopathy (*cmr*) fundus phenotypes. Fundus appearance associated with multifocal retinal dysplasia (MRD; **A**, **B**) compared to canine multifocal retinopathy 3 (*cmr3*; **C**, **D**), canine multifocal retinopathy 1 (*cmr1*; **E**), and a normal reference fundus (**F**). **A**-**B**: MRD: retinal folds with hyporeflective areas. Smaller lesions (arrows) can appear similar to *cmr*, particularly in dogs that have only one or few folds. **C**-**D:** *cmr3*: Multiple brown-gray oval lesions located subretinally (**C**, arrows) that generally are of smaller diameter than the optic disc. Lesions are elevated and are surrounded by a “halo” of presumably clear subretinal fluid (**D**, arrow). **E**. Lesions typical for *cmr1* (arrows). **F**: Normal fundus appearance.

**Figure 2:**
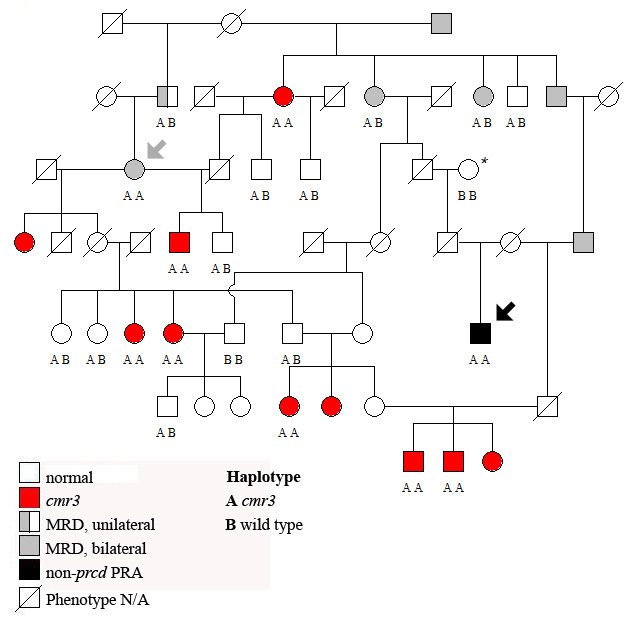
Representative Lapponian herder pedigree analyzed for canine multifocal retinopathy 3 (*cmr3*) mutations. All animals clinically affected with *cmr3* (red) are homozygous for the identified *cmr3* haplotype (AA), as is one individual presenting multifocal retinal dysplasia (MRD; gray, arrow). Additionally, one dog diagnosed with progressive retinal atrophy (PRA) at 8 years of age (black, arrow) shares the disease haplotype; at the same time this animal is related to non-progressive rod-cone degeneration (*prcd*) PRA-affected dogs through one of its ancestors (*; extended pedigree not shown). Abbreviations: AB represents the heterozygote haplotype; BB represents the wild-type haplotype, N/A is not available.

Retinal fundus phenotypes observed in LH and other dog breeds can be related to several different recognized disease variations. The LH breed is known to segregate autosomal recessive *prcd* and a non-*prcd* form of generalized retinal degeneration. Several additional retinal abnormalities also were observed, including acquired post-inflammatory changes, and more commonly a spectrum of fundus changes referred to as multifocal retinal dysplasia (MRD [30]). This latter phenotype is characterized generally by retinal folds and small hyporeflective areas in one or both eyes (Figure 1A,B) that are more frequent in younger animals and become less prominent or disappear in older adults. A subset of LH dogs examined presented with multiple elevated subretinal brown-gray lesions (Figure 1C,D) similar to previously described *cmr* ( [24], Figure 1E). The particular hallmarks of this fundus appearance were bilateral expression, larger lesions as compared to MRD, and clear indication of retinal elevation, often in combination with lighter areas of fluid accumulation next to or around the darker center of the lesions (Figure 1D). Pedigree analysis revealed autosomal recessive inheritance for the *cmr* phenotype (Figure 2) but did not definitively determine if MRD and *cmr* comprise the same or independent genetic traits. To further evaluate the genetic basis for these phenotypes, *BEST1* was considered as a candidate gene for retinal disease in LH and isolated cases from three additional breeds, Karelian beardog, Kuvasz, and Swedish vallhund, diagnosed with MRD.

### *BEST1* mutations

A total of 614 animals from 38 individual breeds (Table 1), including LH, were selected based on (a) results of the above described phenotype evaluation and subsequent outcomes of presented screens (BMD); (b) known involvement with *cmr1* but originating from European populations not previously screened (Dogue de Bordeaux and English mastiff); (c) known mastiff origin (e.g., Italian cane corso and Neapolitan mastiff); (d) control breeds (e.g., Norwegian elkhound and Samoyed).

**Figure 3:**
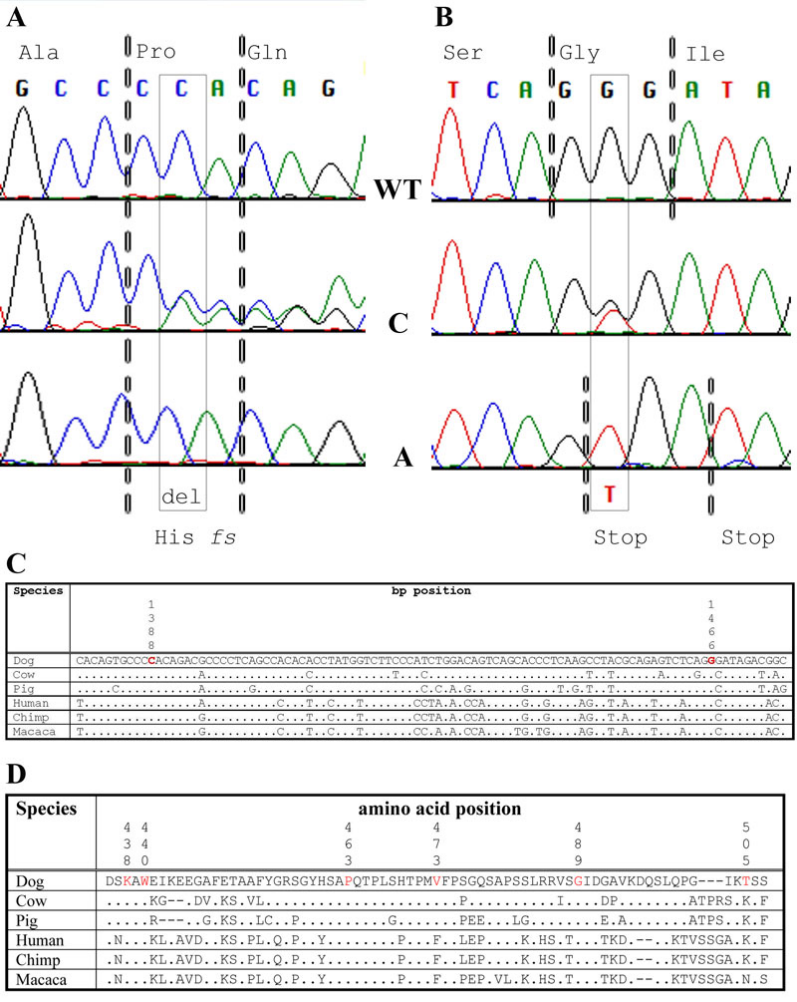
Mutations identified in LH affected with canine multifocal retinopathy (*cmr3*). A C1388del/Pro463*fs* mutation (**A**, boxed) and linked G1466T/Gly489Val nucleotide substitution (**B**, boxed) were identified in canine bestrophin 1 (*cBEST1*) exon 10 of *cmr3*-affected Lapponian herder (LH). Wild-type sequence and resulting amino acids are identified on top (codons are separated by dotted lines), while the mutations and resulting amino acid changes are noted on the bottom. Note that the Gly489Val substitution leads to a stop codon within the Pro463*fs* altered reading frame. The carrier sequence in the middle is heterozygous for both changes. WT=wild type, C=carrier, A=*cmr3* affected. **C**: Conservation of the nucleotide sequence between species. **D**: Comparison of partial bestrophin exon 10, amino acid 436 to 507 between different species demonstrates the conservation of identified variants. Positions 438, 440, 463, 473, 489, and 505 are highlighted in red. Dog=*Canis familiaris*, Cow=*Bos taurus*, Pig=*Sus scrofa*, Human=*Homo sapiens*, Chimp=*Pan troglodytes*, Macaca=*Macaca fascicularis*. “.”=position identical to Dog.

Initially, affected animals from selected breeds with typical *cmr* changes and three LH that were obligate *cmr* carriers (Table 1) had the complete *cBEST1* coding region and corresponding exon–intron boundaries sequenced. Several known and novel (NCBI ss250608388-ss250608389, ss250608394-ss250608401) polymorphisms were identified in these samples (Table 2). Of these, two coding changes that differ from the WT *cBEST1* sequence were found homozygous in affected LH; a deletion at nucleotide position 1,388 of the open reading frame (Figure 3A; NCBI ss250608397), and a substitution at nucleotide position 1,466 (Figure 3B; NCBI ss250608399). The C1388del results in a frame shift (Pro463*fs*) introducing a new stop codon at amino acid 490. The G1466T substitution by itself leads to a conservative change in the amino acid sequence (Gly489Val), which is predicted to change the protein function with only marginal significance (SIFT p=0.05; Table 3). In combination with the C1388del, however, the G1466T substitutions results in an additional stop codon at amino acid position 489 within the shifted reading frame (Gly489X). Since the mutations have only been found in complete linkage disequilibrium, we conclude that the combination of changes results in the disease we now refer to as *cmr3*. Notably, both positions appear highly conserved in the *BEST1* gene of different species at the nucleotide (Figure 3C) and amino acid level (Figure 3D).

**Table 3:** Dog breeds associated with *cBEST1* sequence variations.

**Breed**	**CFA18 bp**	**variant**	**aa change**	**disease**	**Significance (p)**
Bernese mountain dog	57500034	A1313G*	Lys438Arg	-	0.44
	57500027	G1320T*	Trp440Cys	-	0.02
	57499929	T1418G*	Val473Gly	-	0.02
Dogue de Bordeaux	57507861	C73T	R25X	*cmr1*	stop
English mastiff	57507861	C73T	R25X	*cmr1*	stop
Italian cane corso	57507861	C73T	R25X	*cmr1*	stop
Jämthund	57499834	A1513T*	Thr505Ser	*-*	0.75
Lapponian herder	57499959	C1388del	Pro463fs	*cmr3*	frame shift
	57499881	G1466T	Gly489Val	*cmr3*	0.05
Norfolk terrier	57500028	G1319T*	Trp440Leu	-	0.67

New sequence alterations were identified in cBEST1 exon 10 in Bernese mountain dog, Jämthund, Lapponian herder, and Norfolk terrier breeds. Three dog breeds from Italian kennels (Dogue de Bordeaux, English mastiff, Italian cane corso) were found to carry the cmr1 allele [24] in concordance with their mastiff-related background, while the two mutations in the Lapponian herder segregate with an independent disease form, termed cmr3. Significance of probability for missense mutations to alter protein function was calculated using SIFT (column six; Significance [p]). *Changes were observed in heterozygote state only.

The three sequenced obligate LH carrier animals were homozygous for the complete *cBEST1* coding region, with the exception of the above-mentioned mutations and two polymorphisms in introns 2 and 3 (Table 2, #2 and #3; SNP 57,507,851 [BICF2P299043] [27]; SNP 57,506,423 [24]). Although both mutations in exon 10 were found to be linked to each other and the *cmr* phenotype, the intronic polymorphisms were dissociated with the mutations or disease status (data not shown) and therefore cannot predict disease status in the breed.

Focusing on *cBEST1* exon 10, sequence of this exon was obtained from samples of an additional 21 breeds reported to segregate retinopathies and/or unspecified retinal changes (Table 1). None of the investigated animals carried the *cmr3* mutation alleles, but five additional sequence alterations were identified (Table 2 and Table 3).These were analyzed for their potential deleterious effects using SIFT. Two conservative changes in one individual each of the Norfolk terrier and Jämthund breeds were not predicted to impact protein function (p=0.67 and 0.75). Similarly, a Lys438Arg substitution identified in two heterozygous BMD does not affect a critical position in the bestrophin protein (p=0.44) even though this amino acid is highly conserved between species (Figure 3D). The remaining two mutations, Trp440Cys and Val473Gly, have high potential to alter molecular properties (p=0.02 in each case). Both were found in the heterozygous state in BMD at an allele frequency of less than 1% (0.5 and 0.1%, respectively, based on 392 individual dogs), and no homozygous-affected animal has been identified thus far.

Subsequent genotyping of the *cmr1*, *cmr2*, and *cmr3* mutations confirmed segregation of *cmr1* in three mastiff-related breeds from Italian kennels: the Dogue de Bordeaux, English mastiff, and Italian cane corso (Table 3).

### Genotype–phenotype correlation

The two novel *cmr3* mutations found exclusively in the LH breed were in complete linkage equilibrium and homozygous in all animals diagnosed with the *cmr* phenotype (Table 4). Pedigree analysis further supported segregation of the mutations with the *cmr* phenotype (Figure 2). None of the individuals with either post-inflammatory lesions or a normal fundus were affected. However, a single animal initially diagnosed with MRD but not *cmr* also carried the *cmr3* alleles on both chromosomes (Figure 2, gray arrow), as did another animal diagnosed with generalized retinal degeneration (PRA; Figure 2, black arrow). Some animals affected with MRD (Figure 2, gray) were heterozygous for the *cmr3* mutations, but half of the MRD-affected cases typed normal at the *cmr3* loci (Table 4), supporting a disparate genetic basis. It has to be pointed out that the *cmr3*-affected individual diagnosed with PRA is related to non-*prcd* PRA-affected cases that do not carry the *cmr3* alleles homozygously. A common ancestor between these animals (Figure 2, *) indicates that the observed retinal degeneration could be a result of non-*prcd* PRA and mask expression of typical *cmr* fundus changes based on the severe alterations to the retina.

**Table 4:** Lapponian herder phenotype-genotype association.

		***cmr3* genotype**		**Age of diagnosis**	
**Phenotype**	**Total**	**normal**	**carrier**	**affected**	**average years**
normal	31	18	13	0	3–12
MRD	8	4	3	1	2–11
PInf	3	2	1	0	3.0
*cmr*	8	0	0	8	1.8
PRA	4	1	2	1	7.25
Total	54	25	19	10	

Fundus exams of Lapponian herder (LH) identified multifocal retinal dystrophy (MRD), post-inflammatory lesions (PInf), canine multifocal retinopathy (*cmr*), and generalized progressive retinal atrophy, not associated with *prcd* (PRA). Identified *cmr3* mutations segregate with *cmr* (shaded), with two exceptions in other phenotypes (details in text). Most notably, the average age of onset for disease is less than 2 years of age, while PRA typically occurs at a later age. Most variation of disease onset and expression occurs with MRD.

## Discussion

In the present study, a total of 614 dogs from 38 breeds were examined for published *cmr* mutations (*cmr1* and *cmr2*) and then evaluated for the presence of previously unidentified mutations resulting in a *cmr*-like phenotype. Our results (Table 1) confirm segregation of *cmr1* in three mastiff-related breeds from Italy: Dogue de Bordeaux, English mastiff, and Italian cane corso (Table 1). The presence of the *cmr1* allele in the mastiff population is independent of their geographic origin, which dates the original mutation event back to the beginning of the development of this distinct cluster of dog breeds [31]. The lack of the mutation in the Argentinean, Neapolitan, Pyrenean, and Spanish mastiffs is likely due to the small number of available samples examined rather than the exclusion of the disease from these breeds. No additional breeds were found to carry *cmr1* alleles at this point, even when screened animals exhibited a disease phenotype highly compatible with the disease. We therefore conclude that *cmr1* is generally present in mastiff-related breeds. On the other hand, *cmr2* is likely limited to the Coton de Tulear, a breed originating in Madagascar under the strict control of the royal house and isolated for many generations before distribution of a limited breeding stock around the world over the past 35 years. Most notably we have identified a novel *cmr* mutation, *cmr3,* which was observed selectively in LH. Originally used as reindeer-herding dogs by the Sami people of Northern Europe, the breed is thought to have been recreated after a population bottle neck during the Second World War. Although breeding records indicate admixture with other local breeds, including the Karelian beardog, the mutation may either have occurred or been preserved only in those lines leading to the modern dog breed now constituting the LH.

Even though *cmr* and MRD are distinct and separable retinal disorders in dogs, they share enough clinical similarities that could make specific diagnosis difficult. To this end, individuals from four breeds (Table 1), diagnosed with either MRD (Karelian beardog, Kuvasz, Swedish vallhund) or *cmr*-compatible fundus changes (LH), were screened for mutations in the *cBEST1* coding region. Two novel sequence alterations (C1388del/Pro463*fs*, G1466T/Gly489Val) in exon 10 segregated only with the LH *cmr3* (Figure 2). The C1388del microdeletion (Figure 3A) initiates a Pro463*fs* frame shift that results in a stop codon at amino acid 490. To date, the G1466T/Gly489Val substitution has not been observed without the upstream C1388del microdeletion, and the resulting inherent pathogenic effect has therefore not yet been established. Within the context of the upstream mutated reading frame, the G1466T mutation coded for an additional Gly489X stop codon (Figure 3B). These findings strongly indicate that the identified mutations shorten the 585-amino acid bestrophin to a 488-amino acid protein with an altered C-terminus.

The amino acid position and translational consequence of the *cmr3* mutations are distinctly different from the *cmr1* stop mutation (no protein) and the *cmr2* missense mutation (mislocalization; Guziewicz et al., submitted), all resulting in highly similar phenotypes (Figure 1). The *cmr3* mutations likely will not affect bestrophin targeting to the basolateral membrane of the retinal pigment epithelium, but protein function might be impacted by the loss of interaction between the bestrophin C and N-terminus [32]. The bestrophin C-terminus has also been implicated in the activation and regulation of the channel function [33,34], even though most predicted domains are located upstream of the *cmr3* mutations. Additionally, participation in channel multimerization [35] and direct involvement of the C-terminus in the activation or regulation by Ca^2+^ and other physiologic processes has been proposed [36]. However, these predictions are mainly based on hypothesized localization of phosphorylation sites or binding sites for kinases, such as protein kinase A or extracellular signal-regulated kinases (Erk) [37]. Since few human *BEST1* mutations are located within the C-terminal part of bestrophin [7], the *cmr3* model provides initial clinical evidence for a functional role of the *BEST1* C-terminus. Thus, the identification of a third nonallelic form of *cmr* will further contribute to the investigation of *BEST1* disease mechanisms relevant to canines and humans.

*BEST1* exon 10 was sequenced in a larger number of breeds (Table 1) and presented an additional five new sequence alterations (Table 3). Two of these, Thr505Ser and Trp440Leu, were found in the heterozygous state in a single Jämthund and Norfolk terrier, respectively. Clinical reports for these animals did not indicate any retinal changes, and no other individuals from the breeds were available for genotyping. More importantly, both sequence changes are conservative and are not expected to impact protein function [28,29]. Thus, they are not considered to be associated with retinal disease and were not investigated further. Surprisingly, three independent substitutions were present in the BMD breed. Two of these (Trp440Cys and Val473Gly) are likely deleterious (Table 3) and located in a region that is conserved between species (Figure 3D), but neither was found homozygous in an animal with retinal disease. Because of the low allele frequency (< 1%) of these two potential mutations, it is likely that very few if any affected animals will occur in the breed. Hence, the contribution of these mutations to disease risk cannot be evaluated without further elucidating the functional properties of the *BEST1* C-terminus.

Pedigree analysis of *cmr3* supports autosomal recessive inheritance with the predominant clinical appearance similar to *cmr* (Table 4, Figure 2). The partial overlap in phenotype with *prcd* [22], MRD, and non-*prcd* PRA, however, complicates the characterization of the potential phenotypic variation for each individual trait. Above all, some similarities in the appearance of the fundus lesions in MRD and *cmr* (Figure 1) and lack of a clear definition of the MRD phenotype [30] can raise questions with cases that develop either atypical or only few lesions. In strict contrast to MRD, the *cmr* phenotype always presents bilaterally. Nevertheless, several *cmr3* heterozygote animals exhibit a bilateral MRD phenotype, as does one animals homozygous for the mutations (Figure 2, gray arrow). The latter was diagnosed at 7 years of age with minor changes in both eyes consistent with MRD. Earlier exams list no retinal abnormalities at 1.5 years of age, and multiple “rosettes” in the right eye at 5 years of age. Another *cmr3*-affected animal (Figure 2, black arrow) was diagnosed with generalized retinal degeneration during the first available fundus exam at 8 years of age. Three additional animals with non-*prcd* PRA shared common ancestry with this individual; two of these were carriers and one was normal for the *cmr3* mutations. Thus, the observed phenotype could be the consequence of non-*prcd* PRA segregating in the breed. Without early clinical data, we cannot determine whether *cmr*-related retinal changes were present at an earlier time point.

Longitudinal studies of several *cmr3*-affected animals suggest that fluctuations in the appearance and presence of multifocal lesions can occur. This includes the age of onset (between 9 months and 2 years) as well as extent and progression of lesions; in fact some of the individual *cmr* lesions can disappear over time. The age of onset was observed as early as 11 weeks for *cmr1* [38,39] and 15 weeks for *cmr2* [40], with little or no progression of the lesions beyond 1 year of age. However, diminished electroretinograms suggest that Cotons affected with *cmr2* develop retinal degeneration that can be more extensive than the focal lesion [40]. This is caused by the resolution of the detachment resulting in hypertrophic- and hyperplastic-pigmented retinal epithelium and focal outer retinal thinning. In some older animals with extensive lesions, confluence of these areas can lead to a generalized retinal degeneration. The degenerative process occurs earlier in Great Pyrenees (*cmr1*) and is more likely to result in focal rather than generalized retinal degeneration [38,39]. Additionally, the lower life expectancy of this breed could prevent observing advanced disease stages. However, at least one *cmr1* case with resulting generalized retinal degeneration has been observed clinically and confirmed histopathologically (Bruce Grahn, DVM, Professor of Veterinary Ophthalmology and Associate Dean, Western College of Veterinary Medicine, University of Saskatchewan, Saskatoon, Canada). Association of *BEST1* missense mutations with the human PRA equivalent Retinitis Pigmentosa has recently been established [41]. Although there is no current evidence that *cmr* causes PRA in the investigated dog models, we suggest that generalized retinal degeneration is a potential rare outcome of the disorder and can mimic a PRA phenotype in older animals.

**Figure 4:**
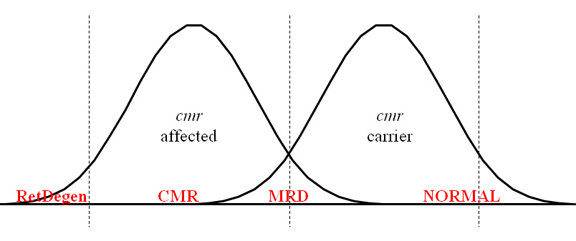
A proposed working hypothesis for canine multifocal retinopathy (*cmr*) genotype–phenotype correlation. *cmr*-affected animals typically develop multifocal lesions, but generalized retinal degeneration (RetDegen) may occasionally be seen in a small number of older dogs as a consequence of the disease. In short, only one animal affected with canine multifocal retinopathy 3 (*cmr3*) was observed in the current study with retinal degeneration; we cannot exclude that this case may have resulted from a mutation at a non-*cmr3* locus because related dogs, normal or carrier for the *cmr3* mutations, were also diagnosed with RetDegen. However, similar consequences have been observed for *cmr1* and *cmr2* (see discussion for details). Few or no animals heterozygous for *cmr3* are expected to exhibit clinical disease, while it cannot be excluded at this point that more susceptible individuals may present a mild disease equivalent to multifocal retinal dysplasia (MRD). The later phenotype could also be observed in some homozygous-affected dogs, although it is not clear whether this is a consequence of incorrectly interpreted fundus appearance or truly signifies the potential of the phenotypic spectrum related to *cmr*. Follow-up long-term studies will further elucidate the validity of the working model.

Based on our current findings, we propose the following hypothetical working model for *cmr3* genotype–phenotype correlations (Figure 4): (a) the majority of *cmr3* homozygous-affected dogs have a typical *cmr* phenotype (Figure 1C,D); (b) at the extremes, a very small number of animals can develop a more subtle phenotype indistinguishable from MRD or generalized retinal degeneration phenotypically identical to PRA; (c) carriers for *cmr3* are asymptomatic, although a few particularly susceptible individuals can develop MRD, possibly using a similar mechanistic basis as autosomal dominant *BEST1*-related human disorders. To test this hypothesis we will need to characterize a larger portion of the population at risk in long-term molecular and clinical studies. Indeed, the establishment of an accurate classification system will aid in the development of categorized information from diagnoses of less defined retinal changes or MRD [30] and would be further simplified by the identification of other loci responsible for MRD or PRA. For this it seems critical to clinically examine dogs at risk between 1 and 2 years of age and provide yearly follow-up data to assess phenotypic variation. Along with the examination, we recommend that photographic documentation be performed using a combination of wide-field and higher resolution images of both eyes; the exact methods have previously been published [42]. Detailed descriptions for canine retinal disorders would enhance our ability to correctly diagnose inherited and acquired diseases, to separate multiple genetic traits in individual breeds, and ultimately to scrutinize *cmr* genotype–phenotype associations.

In summary, the currently identified mutations in *cBEST1* indicate a significant contribution of these variations to retinal disease phenotypes, comparable to the human ortholog. Continued efforts to follow and characterize *cmr* mutations will certainly lay the foundation for understanding phenotype variation and modification of bestrophinopathies as well as promoting the development of new therapies.
